# The structure of 9-(3-bromo-6-chloro-2-hy­droxy­phen­yl)-10-(2-hy­droxy­ethyl)-3,6-diphenyl-3,4,5,6,7,9-hexa­hydro-2*H*-acridine-1,8-dione

**DOI:** 10.1107/S2056989018010873

**Published:** 2018-08-14

**Authors:** Antar A. Abdelhamid, Farouq E. Hawaiz, Alaa F. Mohamed, Shaaban K. Mohamed, Jim Simpson

**Affiliations:** a Chemistry Department, Faculty of Science, Sohag University, Sohag, Egypt; b Chemistry Department, College of Education, Salahaddin University-Hawler, Erbil, Kurdistan Region, Iraq; c National Organization for Drug Control and Research (NODCAR), Giza, Egypt; d Chemistry and Environmental Division, Manchester Metropolitan University, Manchester M1 5GD, England, Chemistry Department, Faculty of Science, Minia University, 61519 El-Minia, Egypt; e Department of Chemistry, University of Otago, PO Box 56, Dunedin, New Zealand

**Keywords:** crystal structure, deca­ahydro­acridine, hydrogen bonds, halogen bonds, C—Br⋯π(ring) contacts

## Abstract

In the structure of the title compound C_33_H_29_BrClNO_4_, (I), the hexa­hydro-2*H*-acridine ring system has a hy­droxy­ethyl substituent on the N atom and a 3-bromo-6-chloro-2-hy­droxy­phenyl substituent on the central C atom at the 9-position. An unusual feature of the mol­ecule is that the substituents at the 3- and 5-positions of the outer cyclo­hexenone rings are phenyl rings rather than the more common dimethyl substituents. C atoms on both of the cyclo­hexenone rings are disordered over two sites. In the crystal structure, O—H⋯O, C—H⋯O and C—H⋯π(ring) hydrogen bonds combine with an Br—O and unusual C—Br⋯π(ring) halogen bonds to generate a three dimensional network with mol­ecules stacked along the *a*-axis direction.

## Abstract 2

The structure of a deca­ahydro­acridine derivative with phenyl substituents at the 3- and 5-positions of the cyclo­hexenone rings is reported. An extensive range of O—H⋯O, C—H⋯O hydrogen bonds augmented by C—H⋯π(ring) hydrogen bonds an O⋯Br halogen bond and an unusual Br⋯π(ring) contact stabilizes the crystal packing.

## Chemical context   

Acridine derivatives form an important class of heterocycles containing nitro­gen with a broad range of pharmaceutical properties. These include compounds that are used as anti-infammatory (Chen *et al.*, 2002 ▸), anti-cancer (Gamega *et al.*, 1999 ▸), anti-microbial (Kaya *et al.*, 2011 ▸), anti-tubercular (Aly & Abadi 2004 ▸; Tripathi *et al.*, 2006 ▸), anti-parasitic (Di Giorgio, *et al.*, 2005 ▸), anti-malarial (Kumar *et al.*, 2009 ▸; Tomar *et al.*, 2010 ▸), anti-viral (Gupta & Jaiswal, 2010 ▸; Tonelli *et al.*, 2011 ▸) and fungicidal agents (Srivastava & Nizamuddin, 2004 ▸). Furthermore, acridines are used as dyes, fluorescent materials for the visualization of biomolecules and in laser technologies (Niknam & Damya, 2009 ▸). In this context we report here the synthesis and crystal structure of the title acridine derivative.
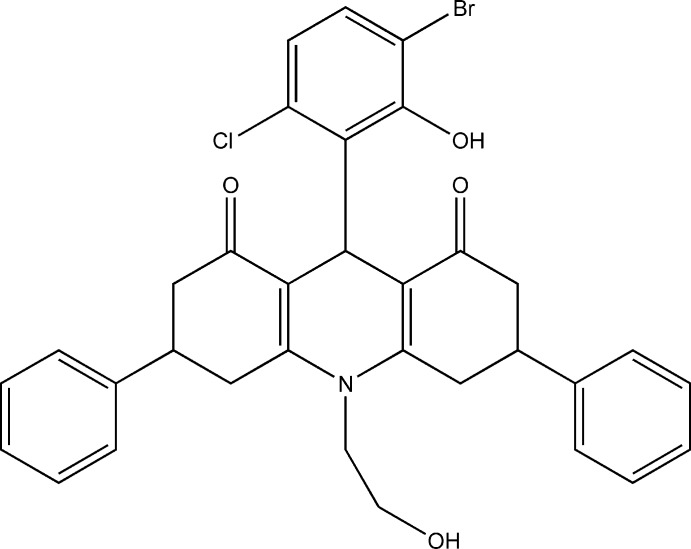


## Structural commentary   

**Figure 1:**
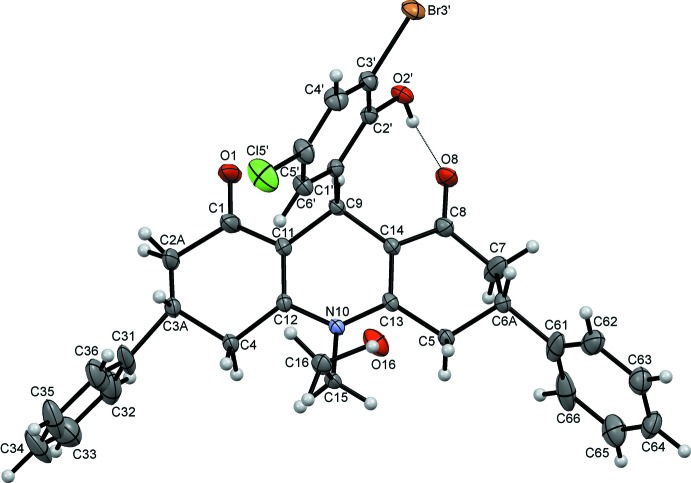
The structure of (I) with ellipsoids drawn at the 50% probability level. For clarity only the major disorder components of the two cyclo­hexenone rings are shown. An intra­molecular hydrogen bond is drawn as a dashed line.

The title compound (I), consists of a hexa­hydro-2*H*-acridine ring system made up of a central di­hydro­pyridine ring with an N-bound 2-hy­droxy­ethyl substituent flanked by two cyclo­hexenone rings that carry phenyl substituents in the 3- and 5-positions, respectively (Fig. 1 ▸). The central C9 atom bears a 3-bromo-6-chloro-2-hy­droxy­phenyl substituent and the O2′ hy­droxy group forms an intra­molecular hydrogen bond to the adjacent O8 carbonyl oxygen enclosing an *S*(8) ring. The C2 and C3 atoms of one cyclo­hexenone are disordered over two sites as is the C6 atom of the corresponding cyclo­hexenone. Their occupancy ratios refine to 0.521 (10):0.479 (10) for C2,C3 and 0.746 (9):0.254 (9) for C6. Only details of the major disorder components will be considered here. The central C9,N10,C11–C14 ring adopts a half-chair conformation and is inclined to the adjacent C1–C4,C11,C12 and C5–C8,C13,C14 rings at angles of 7.11 (18) and 21.64 (10)°, respectively, so the hexa­hydro-2*H*-acridine unit is far from planar. The 3-bromo-6-chloro-2-hy­droxy­phenyl ring subtends an angle of 84.39 (6)° to this central ring. The C1–C4,C11,C12 ring is best described as a severely flattened boat while the C5–C8,C13,C14 system is in a distorted half-chair conformation. The phenyl substituents on these outer cyclo­hexenone rings are inclined to their parent rings at angles of 76.87 (12)° for C31–C36 and 86.27 (8)° for C61–C66. The N-bound 2-hy­droxy­ethyl substituent points away from the convex face of the hexa­hydro-2*H*-acridine system as does the 3-bromo-6-chloro-2-hy­droxy­phenyl substituent.

## Supra­molecular features   

**Figure 2:**
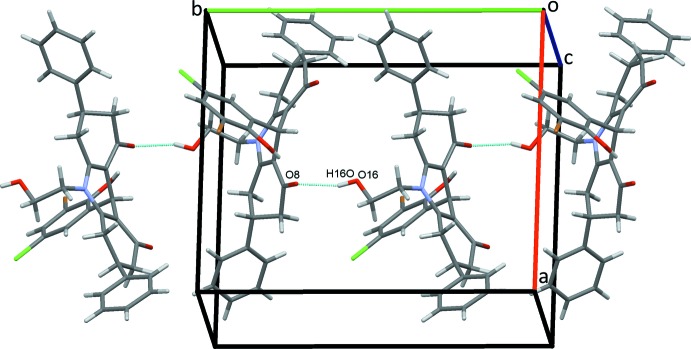
*C*(9) chains of mol­ecules of (I) along *b*. In this and subsequent figures, hydrogen bonds are drawn as dashed lines.

**Figure 3:**
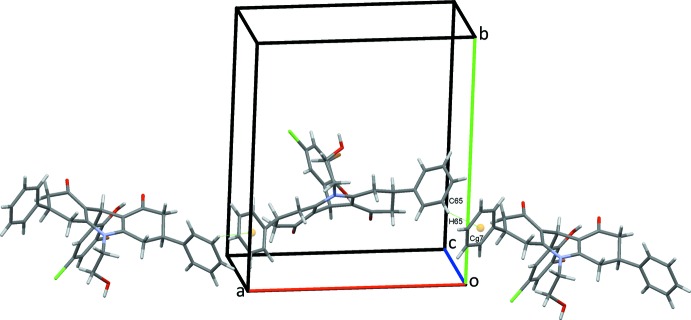
Chains of mol­ecules of (I) along *a*. C—H⋯π contacts are shown as dotted green lines with ring centroids shown as coloured spheres.

**Figure 4:**
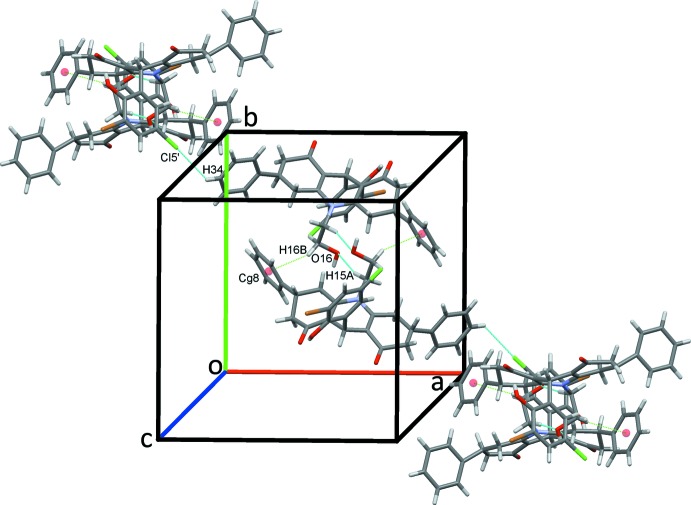
Double chains of mol­ecules of (I) along the *ab* diagonal.

**Figure 5:**
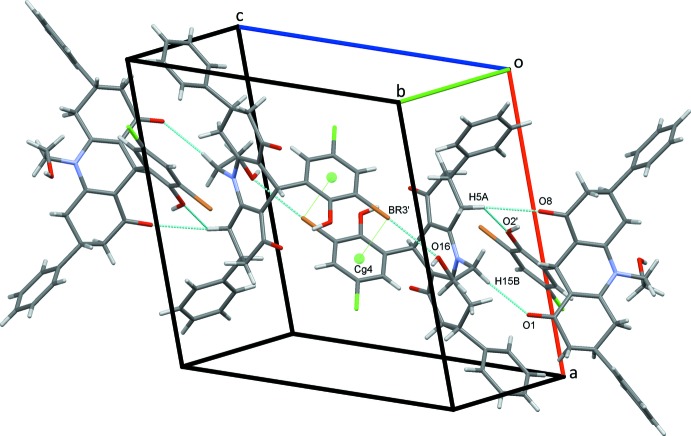
Chains of mol­ecule of (I) formed by C—H⋯O hydrogen bonds, C—Br⋯π and O⋯Br contacts, dotted green lines.

**Figure 6:**
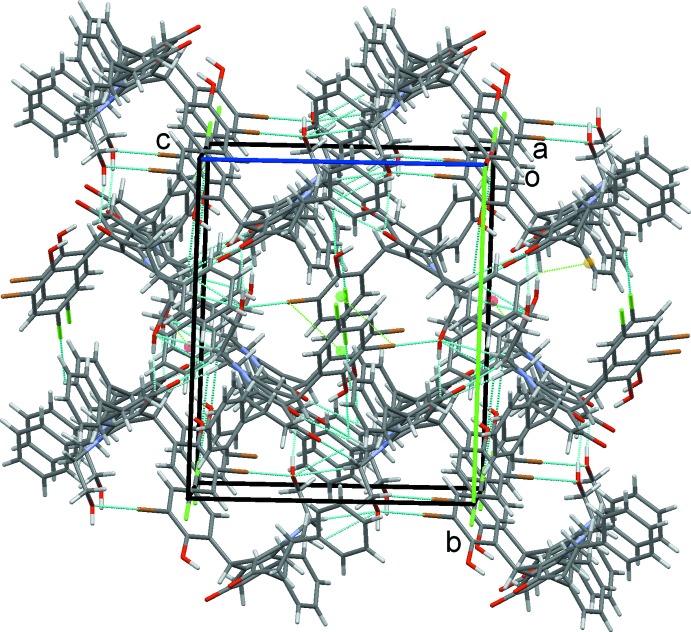
Overall packing of (I) viewed along the *a*-axis direction.

The crystal structure of (I) is supported by a full range of classical and non-classical hydrogen bonds and C—H⋯π(ring) contacts, together with an inter­molecular O⋯Br halogen bond and an unusual C—Br⋯π(ring) contact. Classical O16—H16*O*⋯O8 hydrogen bonds, Table 1 ▸, form *C*(9) chains along the *b-*axis direction, linking the mol­ecules in a head-to-tail fashion, Fig. 2 ▸. Chains also form along the *a-*axis direction through C65—H65⋯*Cg*7 contacts, Fig. 3 ▸, Table 1 ▸. C15—H15*A*⋯O16 hydrogen bonds form inversion dimers that enclose 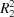
(8) rings and are strengthened by C16—H16⋯*Cg*8 inter­actions. Adjacent dimers are linked by C34—H34⋯Cl5′ hydrogen bonds, forming double chains of mol­ecules along the *ab* diagonal, Fig. 4 ▸. The extensive series of contacts is completed with inversion dimers that also form through O16⋯Br3′^v^ halogen bonds [O⋯Br = 3.0308 (18) Å; symmetry code: (v) 1 − *x*, 1 − *y*, 1 − *z*] (Cavallo *et al.*, 2016 ▸; Chifotides & Dunbar, 2013 ▸) and are supported by unusual C3′—Br3′⋯*Cg*4^v^ contacts [Br3′⋯*Cg*4 = 3.6991 (10) Å, C3′—Br3′⋯*Cg*4 = 83.89 (7)°; *Cg*4 is the centroid of the C1′–C6′ benzene ring] (Matter *et al.*, 2009 ▸; Shukla *et al.*, 2017 ▸; Andleeb *et al.*, 2018 ▸). Both of these contacts are significantly shorter than the sum of the Br and O radii, 3.42 Å (Bondi, 1964 ▸) and that of the Br radius and an estimated half thickness of the benzene ring, 3.75 Å. The dimers are linked into chains running along the *ac* diagonal by a series of C—H⋯O hydrogen bonds generating 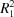
(8) and 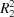
(13) rings, with C5 acting as a bifurcated donor, Table 1 ▸, Fig. 5 ▸. Overall this plethora of inter­molecular contacts combine to generate a complex three-dimensional network with mol­ecules stacked along the *a-*axis direction, Fig. 6 ▸.

## Database survey   

A search of the Cambridge Structural Database (Version 5.39 Nov 2017 with three updates; Groom *et al.* 2016 ▸) for an acridine ring system with a phenyl or substituted benzene ring on the central C9 atom gave 94 hits, 76 of which represented unique occurrences. The majority of these, 58, have two methyl substituents at the 3- and 5-positions of the ring system. However, three instances reveal a pair of methyl substituents on the 3-position only, with the remaining 15 structures having no additional substitution on either of the cyclo­hexenone rings. Inter­estingly, no structures were observed with phenyl substituents at the 3- or the 3- and 5-positions of the hexa­hydro-2*H*-acridine ring system, emphasizing the uniqueness of the structure reported here. Refining the search to structures with CH_2_CH substitution on the acridine N atom reduced the hits to seven, four of which have hy­droxy­ethyl substituents on N10 (Mohamed *et al.*, 2013 ▸; Abdelhamid *et al.*, 2016 ▸, 2014 ▸, 2011 ▸). Only one of the entries has a 2-hy­droxypropyl N10 substituent (Khalilov *et al.*, 2011 ▸), with pairs of methyl substituents on the 3- and 5-positions.

## Synthesis and crystallization   

The title compound was synthesized according to our previously reported method (Mohamed *et al.*, 2013 ▸). Crystals suitable for X-ray diffraction were obtained by the slow evaporation method using ethanol/acetone (5:1) as the solvent mixture. Yield, 79%; m.p. 451 K.

## Refinement   

Crystal data, data collection and structure refinement details are summarized in Table 2 ▸. All H atoms were refined using a riding model with *d*(C—H) = 0.95 Å for aromatic, 0.99 Å for methyl­ene and 1.00 Å for methine H atoms, all with *U*_iso_ = 1.2*U*_eq_(C). The C2 and C3 atoms in the C1–C4,C11,C12 cyclo­hexenone ring and atom, C6, in the corresponding C5–C8,C13,C14 ring are disordered over two positions. Their occupancies were refined to sum to unity with the disordered atoms of the different rings allowed to refine separately. The occupancies converged to ratios of 0.521 (10): 0.479 (10) for C2 and C3 and 0.746 (9): 0.254 (9) for C6. Positions of the hydrogen atoms on adjacent methyl­ene groups and phenyl rings were assigned taking this disorder into account but a somewhat close H15*A*⋯H5*C* contact was still observed. One reflection with *F*_o_ >>> *F*_c_, was omitted from the final refinement cycles.

## Supplementary Material

Crystal structure: contains datablock(s) global, I. DOI: 10.1107/S2056989018010873/ff2154sup1.cif

Structure factors: contains datablock(s) I. DOI: 10.1107/S2056989018010873/ff2154Isup2.hkl

Supporting information file. DOI: 10.1107/S2056989018010873/ff2154Isup3.cml

CCDC reference: 1859007

Additional supporting information:  crystallographic information; 3D view; checkCIF report

## Figures and Tables

**Table 1:** Hydrogen-bond geometry (Å, °) *Cg*7 and *Cg*8 are the centroids of the C31–C36 and C61–C66 phenyl rings, respectively.

*D*—H⋯*A*	*D*—H	H⋯*A*	*D*⋯*A*	*D*—H⋯*A*
O2′—H2′*O*⋯O8	0.85 (3)	1.79 (3)	2.626 (2)	170 (3)
O16—H16*O*⋯O8^i^	0.84 (4)	1.97 (4)	2.782 (2)	163 (4)
C15—H15*A*⋯O16^ii^	0.99	2.68	3.622 (3)	159
C5—H5*A*⋯O8^iii^	0.99	2.69	3.669 (3)	172
C5—H5*A*⋯O2′^iii^	0.99	2.70	3.336 (3)	122
C15—H15*B*⋯O1^iii^	0.99	2.47	3.451 (3)	172
C34—H34⋯Cl5′^iv^	0.95	2.87	3.560 (3)	131
C16—H16*B*⋯*Cg*8^ii^	0.99	2.66	3.529 (3)	147
C65—H65⋯*Cg*7^v^	0.95	2.78	3.648 (4)	152

Symmetry codes: (i) 
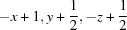
; (ii) 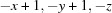; (iii) 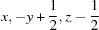; (iv) 
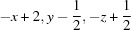
; (v) 
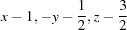
.

**Table 2:** Experimental details

Crystal data	
Chemical formula	C_33_H_29_BrClNO_4_
*M*_r_	618.93
Crystal system, space group	Monoclinic, *P*2_1_/*c*
Temperature (K)	100
*a*, *b*, *c* (Å)	14.5669 (2), 15.4643 (2), 13.4979 (2)
β (°)	107.280 (1)
*V* (Å^3^)	2903.39 (7)
*Z*	4
Radiation type	Cu *K*α
μ (mm^−1^)	3.09
Crystal size (mm)	0.37 × 0.14 × 0.12

Data collection	
Diffractometer	Agilent SuperNova, Dual, Cu at zero, Atlas
Absorption correction	Multi-scan (*CrysAlis PRO*; Agilent, 2014 ▸)
*T*_min_, *T*_max_	0.618, 1.000
No. of measured, independent and observed [*I* > 2σ(*I*)] reflections	23967, 6076, 5714
*R*_int_	0.045
(sin θ/λ)_max_ (Å^−1^)	0.631

Refinement	
*R*[*F* ^2^ > 2σ(*F* ^2^)], *wR*(*F* ^2^), *S*	0.040, 0.101, 1.07
No. of reflections	6076
No. of parameters	402
H-atom treatment	H atoms treated by a mixture of independent and constrained refinement
Δρ_max_, Δρ_min_ (e Å^−3^)	0.66, −0.57

Computer programs: *CrysAlis PRO* (Agilent, 2014 ▸), *SHELXT* (Sheldrick, 2015*a* ▸), *SHELXL2014* (Sheldrick, 2015*b* ▸), *TITAN* (Hunter & Simpson, 1999 ▸), *Mercury* (Macrae *et al.*, 2008 ▸), *enCIFer* (Allen *et al.*, 2004 ▸), *PLATON* (Spek, 2009 ▸), *publCIF* (Westrip 2010 ▸) and *WinGX* (Farrugia 2012 ▸).

## supplementary crystallographic information

### Crystal data

**Table:**

C_33_H_29_BrClNO_4_	*F*(000) = 1272
*M**_r_* = 618.93	*D*_x_ = 1.416 Mg m^−^^3^
Monoclinic, *P*2_1_/*c*	Cu *K*α radiation, λ = 1.54184 Å
*a* = 14.5669 (2) Å	Cell parameters from 16242 reflections
*b* = 15.4643 (2) Å	θ = 4.2–76.6°
*c* = 13.4979 (2) Å	µ = 3.09 mm^−^^1^
β = 107.280 (1)°	*T* = 100 K
*V* = 2903.39 (7) Å^3^	Rectangular plate, pale yellow
*Z* = 4	0.37 × 0.14 × 0.12 mm

### Data collection

**Table:**

Agilent SuperNova, Dual, Cu at zero, Atlas diffractometer	6076 independent reflections
Radiation source: SuperNova (Cu) X-ray Source	5714 reflections with *I* > 2σ(*I*)
Detector resolution: 5.1725 pixels mm^-1^	*R*_int_ = 0.045
ω scans	θ_max_ = 76.7°, θ_min_ = 4.3°
Absorption correction: multi-scan (CrysAlis PRO; Agilent, 2014)	*h* = −18→18
*T*_min_ = 0.618, *T*_max_ = 1.000	*k* = −19→14
23967 measured reflections	*l* = −16→16

### Refinement

**Table:**

Refinement on *F*^2^	0 restraints
Least-squares matrix: full	Hydrogen site location: mixed
*R*[*F*^2^ > 2σ(*F*^2^)] = 0.040	H atoms treated by a mixture of independent and constrained refinement
*wR*(*F*^2^) = 0.101	*w* = 1/[σ^2^(*F*_o_^2^) + (0.0434*P*)^2^ + 3.2091*P*] where *P* = (*F*_o_^2^ + 2*F*_c_^2^)/3
*S* = 1.07	(Δ/σ)_max_ = 0.001
6076 reflections	Δρ_max_ = 0.66 e Å^−^^3^
402 parameters	Δρ_min_ = −0.57 e Å^−^^3^

### Special details

**Table:**

Geometry. All esds (except the esd in the dihedral angle between two l.s. planes) are estimated using the full covariance matrix. The cell esds are taken into account individually in the estimation of esds in distances, angles and torsion angles; correlations between esds in cell parameters are only used when they are defined by crystal symmetry. An approximate (isotropic) treatment of cell esds is used for estimating esds involving l.s. planes.
Refinement. One reflection with Fo >>> Fc was omitted from the final refinement cycles.

### Fractional atomic coordinates and isotropic or equivalent isotropic displacement parameters (Å^2^)

**Table:**

	*x*	*y*	*z*	*U*_iso_*/*U*_eq_	Occ. (<1)
O1	0.75525 (13)	0.16790 (13)	0.44418 (13)	0.0361 (4)	
C1	0.75604 (17)	0.20511 (17)	0.36498 (18)	0.0309 (5)	
C2A	0.8507 (4)	0.2118 (5)	0.3297 (4)	0.0302 (15)	0.521 (10)
H2A1	0.9018	0.2366	0.3886	0.036*	0.521 (10)
H2A2	0.8706	0.1521	0.3196	0.036*	0.521 (10)
C3A	0.8503 (3)	0.2523 (4)	0.2536 (4)	0.0197 (12)	0.521 (10)
H3A	0.8850	0.3059	0.2857	0.024*	0.521 (10)
C2B	0.8203 (4)	0.1718 (4)	0.3031 (5)	0.0198 (12)	0.479 (10)
H2B1	0.8869	0.1711	0.3503	0.024*	0.479 (10)
H2B2	0.8021	0.1109	0.2847	0.024*	0.479 (10)
C3B	0.8224 (4)	0.2130 (4)	0.2140 (5)	0.0240 (15)	0.479 (10)
H3B	0.7874	0.1693	0.1618	0.029*	0.479 (10)
C31	0.9137 (2)	0.2237 (2)	0.1795 (3)	0.0519 (9)	
C32	0.9205 (2)	0.1493 (2)	0.1268 (3)	0.0525 (9)	
H32	0.8802	0.1016	0.1290	0.063*	
C33	0.9860 (3)	0.1432 (2)	0.0700 (3)	0.0552 (8)	
H33	0.9902	0.0916	0.0334	0.066*	
C34	1.0447 (2)	0.2126 (2)	0.0674 (3)	0.0573 (9)	
H34	1.0896	0.2086	0.0289	0.069*	
C35	1.0389 (2)	0.2863 (2)	0.1194 (3)	0.0546 (9)	
H35	1.0792	0.3341	0.1170	0.065*	
C36	0.9734 (2)	0.2917 (2)	0.1762 (3)	0.0499 (8)	
H36A	0.9700	0.3432	0.2132	0.060*	0.521 (10)
H36B	0.9700	0.3432	0.2132	0.060*	0.479 (10)
C4	0.75926 (14)	0.29215 (14)	0.17397 (16)	0.0201 (4)	
H4A	0.7760	0.3489	0.1500	0.024*	0.521 (10)
H4B	0.7374	0.2537	0.1128	0.024*	0.521 (10)
H4C	0.8002	0.3445	0.1879	0.024*	0.479 (10)
H4D	0.7311	0.2870	0.0978	0.024*	0.479 (10)
C5	0.42495 (14)	0.37594 (13)	0.10511 (15)	0.0178 (4)	
H5A	0.4165	0.3450	0.0388	0.021*	0.746 (9)
H5B	0.4335	0.4381	0.0929	0.021*	0.746 (9)
H5C	0.433 (7)	0.370 (7)	0.025 (8)	0.021*	0.254 (9)
H5D	0.422 (8)	0.437 (7)	0.117 (8)	0.021*	0.254 (9)
C6A	0.33431 (18)	0.3641 (2)	0.1396 (2)	0.0195 (9)	0.746 (9)
H6A	0.3356	0.4086	0.1938	0.023*	0.746 (9)
C6B	0.3349 (5)	0.3168 (5)	0.0936 (6)	0.015 (2)	0.254 (9)
H6B	0.3341	0.2706	0.0415	0.019*	0.254 (9)
C61	0.24605 (17)	0.3823 (2)	0.0451 (2)	0.0420 (7)	
C62	0.20200 (18)	0.4592 (2)	0.0484 (2)	0.0396 (6)	
H62	0.2246	0.4951	0.1078	0.047*	
C63	0.12511 (18)	0.48598 (19)	−0.0327 (2)	0.0359 (5)	
H63	0.0953	0.5399	−0.0281	0.043*	
C64	0.09078 (17)	0.43614 (17)	−0.1202 (2)	0.0347 (6)	
H64	0.0377	0.4554	−0.1757	0.042*	
C65	0.13441 (19)	0.35737 (19)	−0.1267 (2)	0.0406 (6)	
H65	0.1118	0.3221	−0.1866	0.049*	
C66	0.21263 (19)	0.33072 (19)	−0.0430 (3)	0.0483 (8)	
H66A	0.2430	0.2770	−0.0466	0.058*	0.746 (9)
H66	0.2430	0.2770	−0.0466	0.058*	0.254 (9)
C7	0.33159 (18)	0.2777 (2)	0.1859 (2)	0.0424 (7)	
H7A	0.3299	0.2323	0.1337	0.051*	0.746 (9)
H7B	0.2729	0.2722	0.2081	0.051*	0.746 (9)
H7C	0.3036	0.2195	0.1671	0.051*	0.254 (9)
H7D	0.2846	0.3109	0.2107	0.051*	0.254 (9)
C8	0.41893 (15)	0.26609 (14)	0.27755 (16)	0.0217 (4)	
O8	0.41215 (11)	0.22515 (11)	0.35542 (12)	0.0253 (3)	
C9	0.59971 (14)	0.28147 (13)	0.35948 (15)	0.0164 (4)	
H9	0.5898	0.2278	0.3964	0.020*	
C11	0.67923 (14)	0.26478 (13)	0.31174 (15)	0.0169 (4)	
C12	0.67918 (13)	0.30368 (13)	0.22213 (15)	0.0165 (4)	
C13	0.51346 (14)	0.34248 (12)	0.18453 (15)	0.0156 (4)	
C14	0.50871 (14)	0.29899 (12)	0.27191 (15)	0.0162 (4)	
N10	0.60071 (11)	0.35389 (11)	0.16665 (13)	0.0159 (3)	
C15	0.61313 (14)	0.41375 (14)	0.08619 (15)	0.0186 (4)	
H15A	0.5504	0.4225	0.0328	0.022*	
H15B	0.6580	0.3879	0.0519	0.022*	
C16	0.65185 (15)	0.49977 (14)	0.13240 (17)	0.0236 (4)	
H16A	0.7049	0.4910	0.1973	0.028*	
H16B	0.6770	0.5327	0.0832	0.028*	
O16	0.57556 (13)	0.54592 (12)	0.15339 (15)	0.0336 (4)	
H16O	0.592 (3)	0.598 (3)	0.156 (3)	0.050*	
C1'	0.62288 (14)	0.35601 (13)	0.43787 (15)	0.0168 (4)	
C2'	0.57556 (14)	0.36228 (13)	0.51490 (15)	0.0179 (4)	
O2'	0.51146 (11)	0.30269 (10)	0.52679 (12)	0.0222 (3)	
H2'O	0.485 (2)	0.274 (2)	0.472 (3)	0.033*	
C3'	0.59615 (15)	0.43193 (14)	0.58369 (17)	0.0223 (4)	
Br3'	0.53322 (2)	0.44056 (2)	0.68754 (2)	0.02632 (9)	
C4'	0.66042 (17)	0.49627 (15)	0.57727 (18)	0.0278 (5)	
H4'	0.6733	0.5436	0.6243	0.033*	
C5'	0.70526 (16)	0.48948 (15)	0.50025 (19)	0.0275 (5)	
Cl5'	0.78601 (5)	0.56959 (4)	0.48926 (6)	0.04541 (18)	
C6'	0.68789 (15)	0.42062 (15)	0.43232 (17)	0.0227 (4)	
H6'	0.7206	0.4172	0.3811	0.027*	

### Atomic displacement parameters (Å^2^)

**Table:**

	*U*^11^	*U*^22^	*U*^33^	*U*^12^	*U*^13^	*U*^23^
O1	0.0387 (9)	0.0496 (11)	0.0255 (8)	0.0246 (8)	0.0179 (7)	0.0171 (8)
C1	0.0314 (12)	0.0418 (14)	0.0240 (11)	0.0176 (10)	0.0152 (9)	0.0099 (10)
C2A	0.023 (2)	0.042 (4)	0.029 (3)	0.017 (3)	0.012 (2)	0.013 (3)
C3A	0.0135 (19)	0.023 (3)	0.024 (2)	0.0011 (18)	0.0071 (17)	0.003 (2)
C2B	0.016 (2)	0.017 (3)	0.030 (3)	0.006 (2)	0.012 (2)	0.007 (2)
C3B	0.018 (2)	0.030 (3)	0.027 (3)	0.006 (2)	0.010 (2)	0.003 (2)
C31	0.0293 (13)	0.075 (2)	0.0634 (19)	0.0323 (14)	0.0320 (13)	0.0481 (17)
C32	0.0248 (12)	0.069 (2)	0.065 (2)	0.0031 (13)	0.0155 (13)	0.0302 (17)
C33	0.0555 (19)	0.060 (2)	0.059 (2)	0.0050 (16)	0.0308 (16)	0.0077 (16)
C34	0.0530 (18)	0.061 (2)	0.080 (2)	0.0205 (16)	0.0544 (18)	0.0245 (18)
C35	0.0339 (14)	0.0482 (17)	0.095 (3)	0.0124 (13)	0.0398 (16)	0.0261 (17)
C36	0.0411 (15)	0.0537 (18)	0.065 (2)	0.0271 (14)	0.0314 (15)	0.0204 (15)
C4	0.0152 (9)	0.0253 (10)	0.0218 (9)	0.0037 (8)	0.0087 (7)	0.0042 (8)
C5	0.0143 (8)	0.0203 (10)	0.0206 (9)	0.0000 (7)	0.0080 (7)	0.0028 (8)
C6A	0.0144 (12)	0.0235 (17)	0.0212 (15)	−0.0021 (10)	0.0062 (10)	0.0004 (13)
C6B	0.012 (3)	0.018 (5)	0.014 (4)	−0.007 (3)	0.000 (3)	−0.003 (3)
C61	0.0148 (10)	0.0588 (18)	0.0492 (16)	−0.0086 (11)	0.0047 (10)	0.0285 (14)
C62	0.0242 (11)	0.0598 (18)	0.0315 (13)	−0.0115 (12)	0.0034 (10)	0.0088 (12)
C63	0.0263 (11)	0.0403 (14)	0.0400 (14)	−0.0004 (10)	0.0083 (10)	0.0025 (11)
C64	0.0193 (10)	0.0414 (14)	0.0371 (13)	−0.0018 (10)	−0.0013 (9)	0.0055 (11)
C65	0.0288 (12)	0.0386 (14)	0.0549 (17)	−0.0125 (11)	0.0134 (12)	−0.0066 (12)
C66	0.0269 (12)	0.0332 (14)	0.093 (2)	0.0073 (11)	0.0301 (15)	0.0277 (15)
C7	0.0212 (11)	0.0646 (19)	0.0358 (13)	−0.0164 (12)	−0.0001 (10)	0.0226 (13)
C8	0.0201 (9)	0.0249 (10)	0.0208 (10)	−0.0044 (8)	0.0074 (8)	0.0022 (8)
O8	0.0240 (7)	0.0309 (8)	0.0232 (7)	−0.0077 (6)	0.0103 (6)	0.0049 (6)
C9	0.0170 (9)	0.0177 (9)	0.0170 (9)	0.0011 (7)	0.0088 (7)	0.0015 (7)
C11	0.0151 (8)	0.0192 (9)	0.0174 (9)	0.0023 (7)	0.0065 (7)	−0.0006 (7)
C12	0.0130 (8)	0.0186 (9)	0.0187 (9)	0.0015 (7)	0.0058 (7)	0.0004 (7)
C13	0.0154 (8)	0.0148 (9)	0.0185 (9)	−0.0001 (7)	0.0081 (7)	−0.0020 (7)
C14	0.0155 (8)	0.0170 (9)	0.0179 (9)	0.0007 (7)	0.0077 (7)	0.0004 (7)
N10	0.0138 (7)	0.0189 (8)	0.0168 (8)	0.0013 (6)	0.0073 (6)	0.0028 (6)
C15	0.0157 (8)	0.0253 (10)	0.0171 (9)	0.0025 (8)	0.0086 (7)	0.0050 (8)
C16	0.0208 (9)	0.0250 (10)	0.0267 (10)	−0.0020 (8)	0.0095 (8)	0.0062 (8)
O16	0.0374 (9)	0.0255 (8)	0.0458 (10)	−0.0031 (7)	0.0247 (8)	−0.0060 (8)
C1'	0.0166 (8)	0.0179 (9)	0.0164 (9)	0.0027 (7)	0.0057 (7)	0.0008 (7)
C2'	0.0176 (9)	0.0191 (9)	0.0178 (9)	0.0036 (7)	0.0067 (7)	0.0025 (7)
O2'	0.0257 (7)	0.0235 (7)	0.0217 (7)	−0.0039 (6)	0.0135 (6)	−0.0014 (6)
C3'	0.0227 (10)	0.0237 (10)	0.0224 (10)	0.0037 (8)	0.0096 (8)	0.0000 (8)
Br3'	0.03193 (14)	0.02757 (14)	0.02364 (13)	0.00358 (9)	0.01467 (9)	−0.00484 (8)
C4'	0.0295 (11)	0.0245 (11)	0.0296 (11)	−0.0018 (9)	0.0093 (9)	−0.0075 (9)
C5'	0.0247 (10)	0.0250 (11)	0.0354 (12)	−0.0065 (9)	0.0131 (9)	−0.0031 (9)
Cl5'	0.0475 (4)	0.0350 (3)	0.0624 (4)	−0.0218 (3)	0.0296 (3)	−0.0141 (3)
C6'	0.0211 (9)	0.0255 (10)	0.0245 (10)	−0.0003 (8)	0.0110 (8)	−0.0004 (8)

### Geometric parameters (Å, º)

**Table:**

O1—C1	1.217 (3)	C61—C66	1.394 (5)
C1—C11	1.464 (3)	C62—C63	1.377 (4)
C1—C2B	1.518 (5)	C62—H62	0.9500
C1—C2A	1.590 (6)	C63—C64	1.374 (4)
C2A—C3A	1.202 (7)	C63—H63	0.9500
C2A—H2A1	0.9900	C64—C65	1.389 (4)
C2A—H2A2	0.9900	C64—H64	0.9500
C3A—C4	1.564 (5)	C65—C66	1.407 (4)
C3A—C31	1.612 (5)	C65—H65	0.9500
C3A—H3A	1.0000	C66—H66A	0.9500
C2B—C3B	1.369 (7)	C66—H66	0.9500
C2B—H2B1	0.9900	C7—C8	1.499 (3)
C2B—H2B2	0.9900	C7—H7A	0.9900
C3B—C4	1.531 (5)	C7—H7B	0.9900
C3B—C31	1.543 (5)	C7—H7C	0.9900
C3B—H3B	1.0000	C7—H7D	0.9900
C31—C32	1.372 (6)	C8—O8	1.256 (3)
C31—C36	1.373 (5)	C8—C14	1.426 (3)
C32—C33	1.393 (4)	C9—C11	1.506 (2)
C32—H32	0.9500	C9—C14	1.516 (3)
C33—C34	1.379 (5)	C9—C1'	1.533 (3)
C33—H33	0.9500	C9—H9	1.0000
C34—C35	1.355 (5)	C11—C12	1.351 (3)
C34—H34	0.9500	C12—N10	1.401 (2)
C35—C36	1.393 (4)	C13—N10	1.374 (2)
C35—H35	0.9500	C13—C14	1.377 (3)
C36—H36A	0.9500	N10—C15	1.478 (2)
C36—H36B	0.9500	C15—C16	1.506 (3)
C4—C12	1.505 (3)	C15—H15A	0.9900
C4—H4A	0.9900	C15—H15B	0.9900
C4—H4B	0.9900	C16—O16	1.418 (3)
C4—H4C	0.9900	C16—H16A	0.9900
C4—H4D	0.9900	C16—H16B	0.9900
C5—C13	1.503 (3)	O16—Br3'^i^	3.0308 (18)
C5—C6A	1.536 (3)	O16—H16O	0.84 (4)
C5—C6B	1.568 (7)	C1'—C6'	1.394 (3)
C5—H5A	0.9900	C1'—C2'	1.411 (3)
C5—H5B	0.9900	C2'—O2'	1.355 (3)
C5—H5C	1.12 (11)	C2'—C3'	1.395 (3)
C5—H5D	0.96 (10)	O2'—H2'O	0.85 (3)
C6A—C7	1.481 (4)	C3'—C4'	1.386 (3)
C6A—C61	1.544 (3)	C3'—Br3'	1.894 (2)
C6A—H6A	1.0000	C4'—C5'	1.386 (3)
C6B—C7	1.398 (8)	C4'—H4'	0.9500
C6B—C61	1.620 (8)	C5'—C6'	1.379 (3)
C6B—H6B	1.0000	C5'—Cl5'	1.745 (2)
C61—C62	1.358 (5)	C6'—H6'	0.9500
			
O1—C1—C11	121.6 (2)	C62—C61—C6B	149.5 (4)
O1—C1—C2B	119.4 (3)	C66—C61—C6B	91.3 (4)
C11—C1—C2B	116.8 (2)	C61—C62—C63	121.2 (3)
O1—C1—C2A	122.0 (2)	C61—C62—H62	119.4
C11—C1—C2A	114.6 (3)	C63—C62—H62	119.4
C3A—C2A—C1	120.4 (4)	C64—C63—C62	121.1 (3)
C3A—C2A—H2A1	107.2	C64—C63—H63	119.4
C1—C2A—H2A1	107.2	C62—C63—H63	119.4
C3A—C2A—H2A2	107.2	C63—C64—C65	119.3 (2)
C1—C2A—H2A2	107.2	C63—C64—H64	120.3
H2A1—C2A—H2A2	106.9	C65—C64—H64	120.3
C2A—C3A—C4	125.5 (4)	C64—C65—C66	118.9 (3)
C2A—C3A—C31	122.0 (4)	C64—C65—H65	120.6
C4—C3A—C31	101.9 (3)	C66—C65—H65	120.6
C2A—C3A—H3A	100.8	C61—C66—C65	120.8 (3)
C4—C3A—H3A	100.8	C61—C66—H66A	119.6
C31—C3A—H3A	100.8	C65—C66—H66A	119.6
C3B—C2B—C1	120.3 (4)	C61—C66—H66	119.6
C3B—C2B—H2B1	107.3	C65—C66—H66	119.6
C1—C2B—H2B1	107.3	C6B—C7—C8	122.8 (3)
C3B—C2B—H2B2	107.3	C6A—C7—C8	109.5 (2)
C1—C2B—H2B2	107.3	C6A—C7—H7A	109.8
H2B1—C2B—H2B2	106.9	C8—C7—H7A	109.8
C2B—C3B—C4	120.9 (4)	C6A—C7—H7B	109.8
C2B—C3B—C31	124.2 (4)	C8—C7—H7B	109.8
C4—C3B—C31	106.8 (3)	H7A—C7—H7B	108.2
C2B—C3B—H3B	99.5	C6B—C7—H7C	106.6
C4—C3B—H3B	99.5	C8—C7—H7C	106.6
C31—C3B—H3B	99.5	C6B—C7—H7D	106.6
C32—C31—C36	118.7 (3)	C8—C7—H7D	106.6
C32—C31—C3B	105.5 (4)	H7C—C7—H7D	106.6
C36—C31—C3B	134.7 (4)	O8—C8—C14	121.73 (19)
C32—C31—C3A	134.0 (3)	O8—C8—C7	119.51 (19)
C36—C31—C3A	107.2 (4)	C14—C8—C7	118.70 (19)
C31—C32—C33	120.6 (3)	C11—C9—C14	107.62 (15)
C31—C32—H32	119.7	C11—C9—C1'	112.62 (16)
C33—C32—H32	119.7	C14—C9—C1'	111.48 (16)
C34—C33—C32	119.4 (3)	C11—C9—H9	108.3
C34—C33—H33	120.3	C14—C9—H9	108.3
C32—C33—H33	120.3	C1'—C9—H9	108.3
C35—C34—C33	120.5 (3)	C12—C11—C1	121.16 (18)
C35—C34—H34	119.7	C12—C11—C9	120.74 (17)
C33—C34—H34	119.7	C1—C11—C9	118.10 (17)
C34—C35—C36	119.6 (3)	C11—C12—N10	120.59 (17)
C34—C35—H35	120.2	C11—C12—C4	122.66 (18)
C36—C35—H35	120.2	N10—C12—C4	116.67 (17)
C31—C36—C35	121.1 (3)	N10—C13—C14	119.85 (18)
C31—C36—H36A	119.5	N10—C13—C5	118.23 (17)
C35—C36—H36A	119.5	C14—C13—C5	121.89 (17)
C31—C36—H36B	119.5	C13—C14—C8	120.01 (18)
C35—C36—H36B	119.5	C13—C14—C9	120.03 (17)
C12—C4—C3B	113.8 (2)	C8—C14—C9	119.87 (17)
C12—C4—C3A	111.0 (2)	C13—N10—C12	119.05 (16)
C12—C4—H4A	109.4	C13—N10—C15	122.07 (16)
C3A—C4—H4A	109.4	C12—N10—C15	118.77 (15)
C12—C4—H4B	109.4	N10—C15—C16	111.18 (16)
C3A—C4—H4B	109.4	N10—C15—H15A	109.4
H4A—C4—H4B	108.0	C16—C15—H15A	109.4
C12—C4—H4C	108.8	N10—C15—H15B	109.4
C3B—C4—H4C	108.8	C16—C15—H15B	109.4
C12—C4—H4D	108.8	H15A—C15—H15B	108.0
C3B—C4—H4D	108.8	O16—C16—C15	107.94 (17)
H4C—C4—H4D	107.7	O16—C16—H16A	110.1
C13—C5—C6A	112.05 (17)	C15—C16—H16A	110.1
C13—C5—C6B	112.7 (3)	O16—C16—H16B	110.1
C13—C5—H5A	109.2	C15—C16—H16B	110.1
C6A—C5—H5A	109.2	H16A—C16—H16B	108.4
C13—C5—H5B	109.2	C16—O16—Br3'^i^	141.43 (14)
C6A—C5—H5B	109.2	C16—O16—H16O	105 (3)
H5A—C5—H5B	107.9	Br3'^i^—O16—H16O	96 (3)
C13—C5—H5C	110 (5)	C6'—C1'—C2'	118.60 (19)
C6B—C5—H5C	100 (5)	C6'—C1'—C9	121.04 (18)
C13—C5—H5D	107 (6)	C2'—C1'—C9	120.31 (17)
C6B—C5—H5D	120 (6)	O2'—C2'—C3'	117.75 (18)
H5C—C5—H5D	106 (8)	O2'—C2'—C1'	123.03 (18)
C7—C6A—C5	111.7 (2)	C3'—C2'—C1'	119.20 (19)
C7—C6A—C61	113.1 (2)	C2'—O2'—H2'O	114 (2)
C5—C6A—C61	107.9 (2)	C4'—C3'—C2'	121.9 (2)
C7—C6A—H6A	108.0	C4'—C3'—Br3'	118.69 (16)
C5—C6A—H6A	108.0	C2'—C3'—Br3'	119.43 (16)
C61—C6A—H6A	108.0	C5'—C4'—C3'	118.0 (2)
C7—C6B—C5	114.4 (5)	C5'—C4'—H4'	121.0
C7—C6B—C61	113.3 (5)	C3'—C4'—H4'	121.0
C5—C6B—C61	102.7 (5)	C6'—C5'—C4'	121.5 (2)
C7—C6B—H6B	108.7	C6'—C5'—Cl5'	119.15 (18)
C5—C6B—H6B	108.7	C4'—C5'—Cl5'	119.32 (18)
C61—C6B—H6B	108.7	C5'—C6'—C1'	120.7 (2)
C62—C61—C66	118.7 (2)	C5'—C6'—H6'	119.6
C62—C61—C6A	114.7 (3)	C1'—C6'—H6'	119.6
C66—C61—C6A	126.5 (3)		
			
O1—C1—C2A—C3A	−176.6 (5)	O1—C1—C11—C9	1.9 (4)
C11—C1—C2A—C3A	−11.9 (8)	C2B—C1—C11—C9	164.9 (4)
C1—C2A—C3A—C4	−8.1 (10)	C2A—C1—C11—C9	−162.9 (4)
C1—C2A—C3A—C31	−146.2 (6)	C14—C9—C11—C12	31.2 (3)
O1—C1—C2B—C3B	−179.8 (5)	C1'—C9—C11—C12	−92.0 (2)
C11—C1—C2B—C3B	16.9 (7)	C14—C9—C11—C1	−148.5 (2)
C1—C2B—C3B—C4	−1.0 (9)	C1'—C9—C11—C1	88.2 (2)
C1—C2B—C3B—C31	143.5 (6)	C1—C11—C12—N10	173.7 (2)
C2B—C3B—C31—C32	85.6 (7)	C9—C11—C12—N10	−6.1 (3)
C4—C3B—C31—C32	−125.8 (4)	C1—C11—C12—C4	−3.0 (3)
C2B—C3B—C31—C36	−106.7 (6)	C9—C11—C12—C4	177.19 (18)
C4—C3B—C31—C36	41.9 (7)	C3B—C4—C12—C11	18.4 (4)
C2A—C3A—C31—C32	55.6 (8)	C3A—C4—C12—C11	−15.3 (4)
C4—C3A—C31—C32	−90.6 (4)	C3B—C4—C12—N10	−158.5 (4)
C2A—C3A—C31—C36	−121.9 (6)	C3A—C4—C12—N10	167.9 (3)
C4—C3A—C31—C36	91.9 (4)	C6A—C5—C13—N10	−176.0 (2)
C36—C31—C32—C33	−0.7 (5)	C6B—C5—C13—N10	145.0 (4)
C3B—C31—C32—C33	169.3 (3)	C6A—C5—C13—C14	6.0 (3)
C3A—C31—C32—C33	−178.0 (3)	C6B—C5—C13—C14	−33.0 (4)
C31—C32—C33—C34	0.3 (5)	N10—C13—C14—C8	−162.73 (19)
C32—C33—C34—C35	−0.1 (6)	C5—C13—C14—C8	15.3 (3)
C33—C34—C35—C36	0.3 (6)	N10—C13—C14—C9	13.7 (3)
C32—C31—C36—C35	0.9 (5)	C5—C13—C14—C9	−168.33 (17)
C3B—C31—C36—C35	−165.5 (4)	O8—C8—C14—C13	179.0 (2)
C3A—C31—C36—C35	178.9 (3)	C7—C8—C14—C13	1.7 (3)
C34—C35—C36—C31	−0.8 (5)	O8—C8—C14—C9	2.6 (3)
C2B—C3B—C4—C12	−15.9 (7)	C7—C8—C14—C9	−174.7 (2)
C31—C3B—C4—C12	−165.8 (3)	C11—C9—C14—C13	−35.1 (2)
C2A—C3A—C4—C12	21.5 (7)	C1'—C9—C14—C13	88.9 (2)
C31—C3A—C4—C12	166.1 (3)	C11—C9—C14—C8	141.31 (19)
C13—C5—C6A—C7	−43.4 (3)	C1'—C9—C14—C8	−94.7 (2)
C13—C5—C6A—C61	−168.4 (2)	C14—C13—N10—C12	15.8 (3)
C13—C5—C6B—C7	34.3 (7)	C5—C13—N10—C12	−162.27 (17)
C13—C5—C6B—C61	157.5 (3)	C14—C13—N10—C15	−167.96 (18)
C7—C6A—C61—C62	130.3 (3)	C5—C13—N10—C15	14.0 (3)
C5—C6A—C61—C62	−105.7 (3)	C11—C12—N10—C13	−20.0 (3)
C7—C6A—C61—C66	−53.9 (4)	C4—C12—N10—C13	156.96 (18)
C5—C6A—C61—C66	70.1 (3)	C11—C12—N10—C15	163.66 (19)
C7—C6B—C61—C62	73.4 (8)	C4—C12—N10—C15	−19.4 (3)
C5—C6B—C61—C62	−50.6 (9)	C13—N10—C15—C16	97.4 (2)
C7—C6B—C61—C66	−116.4 (5)	C12—N10—C15—C16	−86.4 (2)
C5—C6B—C61—C66	119.6 (4)	N10—C15—C16—O16	−75.8 (2)
C66—C61—C62—C63	0.7 (4)	C15—C16—O16—Br3'^i^	80.6 (3)
C6A—C61—C62—C63	176.9 (2)	C11—C9—C1'—C6'	24.5 (3)
C6B—C61—C62—C63	169.5 (6)	C14—C9—C1'—C6'	−96.6 (2)
C61—C62—C63—C64	−0.5 (4)	C11—C9—C1'—C2'	−158.07 (18)
C62—C63—C64—C65	0.1 (4)	C14—C9—C1'—C2'	80.8 (2)
C63—C64—C65—C66	0.2 (4)	C6'—C1'—C2'—O2'	−179.77 (18)
C62—C61—C66—C65	−0.4 (4)	C9—C1'—C2'—O2'	2.7 (3)
C6A—C61—C66—C65	−176.1 (2)	C6'—C1'—C2'—C3'	−0.9 (3)
C6B—C61—C66—C65	−174.8 (3)	C9—C1'—C2'—C3'	−178.40 (18)
C64—C65—C66—C61	0.0 (4)	O2'—C2'—C3'—C4'	−179.7 (2)
C5—C6B—C7—C8	−20.7 (8)	C1'—C2'—C3'—C4'	1.3 (3)
C61—C6B—C7—C8	−138.1 (4)	O2'—C2'—C3'—Br3'	−0.8 (3)
C5—C6A—C7—C8	58.8 (3)	C1'—C2'—C3'—Br3'	−179.80 (15)
C61—C6A—C7—C8	−179.2 (2)	C2'—C3'—C4'—C5'	−0.6 (3)
C6B—C7—C8—O8	−175.2 (5)	Br3'—C3'—C4'—C5'	−179.42 (18)
C6A—C7—C8—O8	143.9 (2)	C3'—C4'—C5'—C6'	−0.7 (4)
C6B—C7—C8—C14	2.2 (6)	C3'—C4'—C5'—Cl5'	179.59 (18)
C6A—C7—C8—C14	−38.7 (3)	C4'—C5'—C6'—C1'	1.2 (4)
O1—C1—C11—C12	−177.9 (2)	Cl5'—C5'—C6'—C1'	−179.13 (17)
C2B—C1—C11—C12	−14.9 (5)	C2'—C1'—C6'—C5'	−0.4 (3)
C2A—C1—C11—C12	17.3 (5)	C9—C1'—C6'—C5'	177.2 (2)

Symmetry code: (i) −*x*+1, −*y*+1, −*z*+1.

### Hydrogen-bond geometry (Å, º)

Cg7 and Cg8 are the centroids of the C31–C36 and C61–C66 phenyl rings, respectively.

**Table:**

*D*—H···*A*	*D*—H	H···*A*	*D*···*A*	*D*—H···*A*
O2′—H2′*O*···O8	0.85 (3)	1.79 (3)	2.626 (2)	170 (3)
O16—H16*O*···O8^ii^	0.84 (4)	1.97 (4)	2.782 (2)	163 (4)
C15—H15*A*···O16^iii^	0.99	2.68	3.622 (3)	159
C5—H5*A*···O8^iv^	0.99	2.69	3.669 (3)	172
C5—H5*A*···O2′^iv^	0.99	2.70	3.336 (3)	122
C15—H15*B*···O1^iv^	0.99	2.47	3.451 (3)	172
C34—H34···Cl5′^v^	0.95	2.87	3.560 (3)	131
C16—H16*B*···*Cg*8^iii^	0.99	2.66	3.529 (3)	147
C65—H65···*Cg*7^vi^	0.95	2.78	3.648 (4)	152

Symmetry codes: (ii) −*x*+1, *y*+1/2, −*z*+1/2; (iii) −*x*+1, −*y*+1, −*z*; (iv) *x*, −*y*+1/2, *z*−1/2; (v) −*x*+2, *y*−1/2, −*z*+1/2; (vi) *x*−1, −*y*−1/2, *z*−3/2.

## References

[bb1] Abdelhamid, A. A., Mohamed, S. K., Khalilov, A. N., Gurbanov, A. V. & Ng, S. W. (2011). *Acta Cryst.* E**67**, o744. 10.1107/S1600536811006969 PMC3051986 21522483

[bb2] Abdelhamid, A. A., Mohamed, S. K. & Simpson, J. (2014). *Acta Cryst.* E**70**, 44–47. 10.1107/S1600536814009556 PMC4158539 25249850

[bb3] Abdelhamid, A. A., Mohamed, S. K. & Simpson, J. (2016). *IUCrData*, **1**, x152425.

[bb4] Agilent (2014). *CrysAlis PRO*. Agilent Technologies, Yarnton, Oxfordshire, England.

[bb5] Allen, F. H., Johnson, O., Shields, G. P., Smith, B. R. & Towler, M. (2004). *J. Appl. Cryst.* **37**, 335–338.

[bb6] Aly, E. I. & Abadi, A. H. (2004). *Arch. Pharm. Res.* **27**, 713–719. 10.1007/BF02980137 15356996

[bb7] Andleeb, H., Khan, I., Bauzá, A., Tahir, M. N., Simpson, J., Hameed, S. & Frontera, A. (2018). *Acta Cryst.* C**74**, 816–829. 10.1107/S2053229618008355 29973421

[bb8] Bondi, A. (1964). *J. Phys. Chem.* **68**, 441–451.

[bb9] Cavallo, G., Metrangolo, P., Milani, R., Pilati, T., Priimagi, A., Resnati, G. & Terraneo, G. (2016). *Chem. Rev.* **116**, 2478–2601. 10.1021/acs.chemrev.5b00484 PMC4768247 26812185

[bb10] Chen, Y. L., Lu, C. M., Chen, I. L., Tsao, L. T. & Wang, J. P. (2002). *J. Med. Chem.* **45**, 4689–4694. 10.1021/jm020102v 12361395

[bb11] Chifotides, H. T. & Dunbar, K. R. (2013). *Acc. Chem. Res.* **46**, 894–906. 10.1021/ar300251k 23477406

[bb12] Di Giorgio, C., De Meo, M., Chiron, J., Delmas, F., Nikoyan, A., Jean, S., Dumenil, G., Timon-David, P. & Galy, J. P. (2005). *Bioorg. Med. Chem.* **13**, 5560–5568.

[bb13] Farrugia, L. J. (2012). *J. Appl. Cryst.* **45**, 849–854.

[bb14] Gamage, S. A., Spicer, J. A., Atwell, G. J., Finlay, G. J., Baguley, B. C. & Denny, W. A. (1999). *J. Med. Chem.* **42**, 2383–2393. 10.1021/jm980687m 10395479

[bb15] Groom, C. R., Bruno, I. J., Lightfoot, M. P. & Ward, S. C. (2016). *Acta Cryst.* B**72**, 171–179. 10.1107/S2052520616003954 PMC4822653 27048719

[bb16] Gupta, H. C. & Jaiswal, V. (2010). *Indian J. Heterocycl. Chem.* **19**, 409–410.

[bb17] Hunter, K. A. & Simpson, J. (1999). *TITAN2000*. University of Otago, New Zealand.

[bb18] Kaya, M., Yıldırır, Y. & Çelik, G. Y. (2011). *Med. Chem. Res.* **20**, 293–299.

[bb19] Khalilov, A. N., Abdelhamid, A. A., Gurbanov, A. V. & Ng, S. W. (2011). *Acta Cryst.* E**67**, o1146. 10.1107/S1600536811013481 PMC3089333 21754454

[bb20] Kumar, A., Srivastava, K., Kumar, S. R., Puri, S. K. & Chauhan, M. S. (2009). *Bioorg. Med. Chem. Lett.* **19**, 6996–6999. 10.1016/j.bmcl.2009.10.010 19879137

[bb21] Macrae, C. F., Bruno, I. J., Chisholm, J. A., Edgington, P. R., McCabe, P., Pidcock, E., Rodriguez-Monge, L., Taylor, R., van de Streek, J. & Wood, P. A. (2008). *J. Appl. Cryst.* **41**, 466–470.

[bb22] Matter, H., Nazaré, M., Güssregen, S., Will, D. W., Schreuder, H., Bauer, A., Urmann, M., Ritter, K., Wagner, M. & Wehner, V. (2009). *Angew. Chem. Int. Ed.* **48**, 2911–2916. 10.1002/anie.200806219 19294721

[bb23] Mohamed, S. K., Akkurt, M., Horton, P. N., Abdelhamid, A. A. & Remaily, M. A. A. E. (2013). *Acta Cryst.* E**69**, o85–o86. 10.1107/S1600536812050222 PMC3588342 23476467

[bb24] Niknam, K. & Damya, M. (2009). *Jnl Chin. Chem. Soc.* **56**, 659–665.

[bb25] Sheldrick, G. M. (2015*a*). *Acta Cryst.* A**71**, 3–8.

[bb26] Sheldrick, G. M. (2015*b*). *Acta Cryst.* C**71**, 3–8.

[bb27] Shukla, R., Khan, I., Ibrar, A., Simpson, J. & Chopra, D. (2017). *CrystEngComm*, **19**, 3485–3498.

[bb28] Spek, A. L. (2009). *Acta Cryst.* D**65**, 148–155. 10.1107/S090744490804362X PMC2631630 19171970

[bb29] Srivastava, A. & Nizamuddin, A. (2004). *Indian J. Heterocycl. Chem.* **13**, 261–264.

[bb30] Tomar, V., Bhattacharjee, G., Kamaluddin, S. R., Rajakumar, S., Srivastava, K. & Puri, S. K. (2010). *Eur. J. Med. Chem.* **45**, 745–751. 10.1016/j.ejmech.2009.11.022 20022412

[bb31] Tonelli, M., Vettoretti, G., Tasso, B., Novelli, F., Boido, V., Sparatore, F., Busonera, B., Ouhtit, A., Farci, P., Blois, S., Giliberti, G. & La Colla, P. (2011). *Antiviral Res.* **91**, 133–141. 10.1016/j.antiviral.2011.05.005 21619897

[bb32] Tripathi, R. P., Verma, S. S., Pandey, J., Agarwal, K. C., Chaturvedi, V., Manju, Y. K., Srivastva, A. K., Gaikwad, A. & Sinha, S. (2006). *Bioorg. Med. Chem. Lett.* **16**, 5144–5147. 10.1016/j.bmcl.2006.07.025 16870429

[bb33] Westrip, S. P. (2010). *J. Appl. Cryst.* **43**, 920–925.

